# EHMTI-0071. Psychological aspects, comorbidity and drugs usage in medication overuse headache patients

**DOI:** 10.1186/1129-2377-15-S1-D30

**Published:** 2014-09-18

**Authors:** R Iannacchero, F Migliazza, A Squillace, G Vescio, A Costa

**Affiliations:** 1 Centre for Headache and Adaptive Disorders, Pugliese-Ciaccio Hospital, Catanzaro, Italy

## Introduction

Medication Overuse Headache (MOH) patients are often treated in multidisciplinary clinical settings.

## Aims

purpose of this study is obtaining preliminary data about MOH prevalence in our headache centre, identifying MOH patients features in terms of medication usage, comorbidity, psychological aspects and level of disability.

## Methods

Clinical records of 89 patients accessing from January 2014 to April 2014 to our headache centre were reviewed and 27 MOH patients (2 males; 25 females) from 23 to 66 years old aged (M=40.63; DS=10.21) records were selected; demographical, clinical and psycho-social information were retrieved. Frequencies and covariance analyses were performed.

## Results

MOH prevalence is 30.33%; migraine monthly frequency is high (M=16.74; DS=6.09); familiarity rate is 55.56%; comorbidity rate is 77.78%; most prevalent comorbidities are psychopathological (51.85%; mood disorders 25.93%; anxiety disorders 18.52%) and metabolical (22.22%); MIDAS mean score is 42.37; Zung-A mean score is 47.41; Zung-D mean score is 46.00; Prevalent personality traits are: evitating (25.93%) and obsessive-compulsive (18.52%);Self efficacy is predominantly low to middle (mode: sometimes); Overused medication are prevalently NSAID (59.26%) and combination analgesics (29.63%) with a average medications usage of 5397,04 mg/month and a mean overuse duration of 63.63 months; migraine frequency negatively correlates with age (p<0.05) and positively correlates with anxiety (p<0.01), depression (p<0.05), overuse (p<0.05); and disability level (p<0.05). Medication overuse positively correlates with disability (p<0.05).

## Conclusions

The study suggest a relationship between medication overuse, health problems comorbidity and psychological aspects such as personality traits, self efficacy cognitions, psychopathological syndromes and disability.

No conflict of interest.

